# The social and economic determinants of suicide in Canadian provinces

**DOI:** 10.1186/s13561-015-0041-y

**Published:** 2015-01-31

**Authors:** João T Jalles, Martin A Andresen

**Affiliations:** 1 Centre for Globalization and Governance, Nova School of Business and Economics, Campus Campolide, Lisbon, 1099-032 Portugal; 2 School of Criminology, Simon Fraser University, 8888 University Drive, Burnaby, BC V5A 1S6 Canada

**Keywords:** Suicide, Socioeconomic, Panel estimation, Endogeneity, C33, E10, I10

## Abstract

**Background:**

In this paper we investigate the causal relationship between suicide and a variety of socioeconomic variables. We use a panel data set of Canadian provinces, 2000 – 2008, and a set of recent panel econometric techniques in order to account for a variety of statistical specification issues.

**Results:**

We find that the social and economic determinants of suicide in Canadian provinces vary across total, male, and female counts (natural logarithms) and rates. We also find that the results vary depending on the econometric method employed. As such, separate analyses for males and females is necessary for a better understanding of the factors that impact suicide (consistent with previous research) and that the choice of statistical method impacts the results. Lastly, it is important to note the particular provinces are driving the results for particular socioeconomic variables.

**Conclusions:**

Such a result, if generalizable, has significant implications for suicide prevention policy.

## Background

One of the most common explanations for (aggregate) suicide behavior is that of societal conditions, most famously associated with Durkheim [1]. Negative changes in societal conditions lead to anomie, a sense of normlessness, that subsequently leads to an increase in suicidal behavior at the individual level. This relationship may also be understood through the theoretical perspective put forth by Hamermesh and Soss [2], who argue that an individual will commit suicide if their perceived happiness (discounted lifetime utility) falls below some threshold. As such, there is some minimum level of happiness (current and future) necessary for individuals to enjoy life and want for it to continue. Consequently, negative life events such as adverse financial circumstances and social isolation, loss of a loved one or interpersonal conflict, and stress caused by working roles may lead to increases in the suicide rate [3-8]. This model may also be extended to explicitly consider human capital: when unemployed, for example, human capital no longer accumulates because on-the-job training ceases. Therefore, the current period’s income decreases, but also next period’s income, leading to an increased risk of suicide [9].^a^ This theoretical perspective has been tested empirically, identifying three primary determinants of suicide: income, unemployment and age [13-16].

Though these are individual-level explanations for the phenomenon of suicide, the crux of Durkheim’s theory is at the level of a society. As such, and reviewed below, there are many studies that have taken these concepts of negative life events and human capital and used proxies in analyses of suicide rates that can be understood within an economics of suicide perspective. Income levels, unemployment rates, divorce rates, and so on, can measure the relative levels of anomie/normlessness over time and space, and are understandable through an economic lens. In this paper, we analyze a panel of 10 Canadian provinces, 2000 – 2008. We use Canadian data because most suicide research is focused on the United States and Europe [17,18]. In our analyses, we consider these socioeconomic determinants on total and gender-based suicide rates to prevent the possibility of confounding results [19].

As surveyed by Marcotte [13] all empirical work on the economics of suicide to date follow the same framework originated by Hamermesh and Soss [2]. They formalized a purely economic model of individual utility maximization to examine the 1970 cross-state suicide rates in the US. Their work, and subsequent studies of suicide rates by economists, established the notion that an individual will commit suicide when the discounted stream of expected utility over this person’s lifetime falls below his/her threshold utility level of living. Within this framework, three main determinants of suicide are identified: income, unemployment and age. Because a higher income level implies greater consumption and satisfaction, it should lead to a lower suicide propensity. This is the line of Henry and Short [20], who argue that economic prosperity can lead to decreases in suicide mortality [21-24]. However, some studies indicate that suicide rates have positive association with income [2,25-28]. Unemployment, on the other hand, should raise the propensity of suicide as it reduces an individual’s expectation of future income (and utility). The positive connection between unemployment and suicide has been established by empirical evidence [2,29-34]. Age is hypothesized to raise suicide tendency because old age increases the maintenance cost of daily life and health thereby dragging down the remaining lifetime utility. Altogether, at the aggregate level, a state or region’s suicide rate is expected to be negatively associated with the corresponding aggregate indicator of income, and positively related to unemployment and age. These three variables are used in the paper, in particular the log of income per capita, the unemployment rate and the old dependency ratio.

As also indicated in Marcotte [13], most of the sociological work on suicide has been dominated by the conceptual work developed by Durkheim [1]. He postulated that societal suicide rates were influenced by social integration and social regulation. From this perspective, several social indicators are hypothesized to have an impact on societal suicide rates and have been extensively studied by sociologists, including divorce, female labor participation, and migration, to which we now turn.

Divorce is believed to cause a reduction in social integration and regulation, as it involves disruption of family and social ties and itself is a deviation from social norms. Because divorce is viewed as a source of individual trauma that conceivably might trigger suicide, a society characterized by a high divorce is expected to have a higher suicide rate [35].

Sociologists generally regard the increasing female participation into the labor force as an important social phenomenon and argue that it will have significant impact on the societal suicide rates. However, they cannot agree on the direction of its effect on suicide rates as it impacts the society in two opposing ways. On the one hand, women working may decrease social integration due to the possible role conflict and stress between men and women arising from participation, and this may lead to higher suicide rates. On the other hand, women working may strengthen their social bonds and integration because labor market participation provides opportunities for women to develop themselves more fully. As a result of this role accumulation and expansion, societal suicide rates could be lower [36]. All in all the effect is ambiguous.

Migration itself is a stressful process and leaving friends and relatives behind also ruptures social relationships and reduces both social integration and regulation with the original community for the migrants. In addition to the uprooting pains, the move also exposes the migrants to social isolation in a new and unfamiliar community. All these are expected to raise the propensity of suicide among maladjusted migrants and increase the community suicide rates. Taylor [37] viewed migration as “both a structural cause of and an individual motivation for suicide”. Lester’s correlational study for the suicide rate and 27 other variables showed that divorce and interstate migration had the highest correlation with suicide [38].

A few more recent studies support the notion that alcohol consumption tends to increase suicide rates [21,22,39,40]. However, the contribution that alcohol consumption in itself makes to suicide risk remains imperfectly understood. As Lester points out, alcohol and suicide may be associated through unobserved factors [41]. Alcoholics exhibit elevated incidences of other psychological episodes (mental disorders, experience the loss of friends, lack of support from society). Thus, high levels of alcohol consumption may be correlated with the above factors increasing the propensity of committing suicide and has its greatest impact on young males.

It has been also argued that inequality may influence suicide rates. Nonetheless, the causal path why one might expect inequality to affect suicide rates is non-trivial. Kawachi et al. argue that communities with low social capital may have high levels of stress, and high violent crimes [42]. Hence, inequality contributes to reduce social integration and increase mortality. Prior cross-sectional or multivariate analyses failed to find the harmful hypothesized effects of income inequality on suicide [38,43,44]. Recent empirical evidence seems to indicate that past studies were likely wrong as inequality adversely affects the health of the population. Neumayer using a panel of 11–16 German states from 1980–2000 finds that a state’s Gini coefficient has a positive but statistically insignificant effect on male and female suicide rates [22]. This finding is in line with cross-country studies examining the relationship between income inequality and aggregate mortality [45].

The Canadian literature on the socio-demographic and socio-economic factors relating to suicide is relatively small compared to that from the United States and the rest of the world, but instructive nonetheless particularly for the current analysis. The Canadian literature in this area is dominated by Frank Trovato and colleague, focusing on the Durkheimian theory of social integration and suicide: as social integration increases (however measured) suicide rates are expected to decrease. The work undertaken by Frank Trovato and colleague focuses on three aspects of social integration: ethnic factors, migration (interprovincial and international), and marital dissolution.

With regard to ethnicity in Canadian suicide, Trovato analyzed eight different immigrant-ethnic groups in 1971 and 1981,^b^ and found that the greater the degree of social assimilation, i.e. a “breakdown” in the ethnic cluster, led to greater suicide rates [46]. However, ethic groups with higher degrees of community cohesiveness had lesser suicide rates such that the “loss” of one form of social integration may be compensated through the establishment of anther form of social integration. Curiously, Trovato found no support for the role of socioeconomic status impacting suicide rates [46].

In the context of international immigration and suicide, there has been a time series analysis [47] and a cross-sectional analysis [48]. Controlling for unemployment and age, Trovato showed the importance of separating males and females in the analysis to avoid obtaining contradictory results, also stated above [47]. He found that 15–34 year old males are quite sensitive to changes in their employment and immigrant status such that they have greater rates of suicide than their female counterparts. Trovato and Jarvis hypothesized that immigrant groups would initially have a greater rate of suicide than the rest of the population, but that difference would decrease as those immigrant groups spent more time in Canada—increased time in the new country would lead to increased social integration [48]. Using the same immigrant-ethnic groups as Trovato [46], Trovato and Jarvis found that increased social integration, as hypothesized, led to lesser suicide rates [48]. Moreover, immigrant groups with Roman Catholic backgrounds had lesser suicide rates. With regard to interprovincial migration, Trovato found that the relationship with suicide is very similar to that of international immigration [49]. Interprovincial migration still led to severing social ties in the home province and Trovato found that interprovincial migrations does lead to greater rates of suicide, but education is a mitigating factor that leads to less rates of suicide [49].

Similar to immigration, marriage dissolution (divorce) is expected to lead to greater suicide rates because of a loss of social integration. Trovato found strong support for this hypothesis after controlling for education, migration, percentage Roman Catholic, and the marriage rate, confirming results based on data from the United States [50]. In a longitudinal analysis, Trovato found a positive relationship between divorce and suicide after controlling for unemployment and female labor market participation rates [51]. Additionally, Trovato found that for young men, unemployment is positively related to suicide and that female labor market participation only has a positive relationship with suicide for males at the national level [51]. Moreover, female labor market participation has a negative relationship with suicide for females, potentially because of the increased social network that labor market participation provides, as discussed above. And in an analysis of individual death records in an attempt to confirm aggregate analyses, Trovato found that the transition to marriage from being single or widowed reduced suicide rates for men more than women, but the transition from divorce to marriage benefitted males and females equally [52].

In the last Canadian-specific analysis of suicide known to the authors that analyzes youth suicide in the context of family integration (divorce), religious integration (any religious affiliation), and unemployment, Trovato found that a lack of religious affiliation is associated with greater rates of suicide, as is divorce for males and females but only in one of the years under analysis. And unemployment is not found to have a statistically significant relationship with suicide rates, contrary too much of the research based in the United States [53].

## Methods

### Data and descriptive statistics

The panel data used contains 10 Canadian provinces^c^ for the years 2000–2008 were obtained from Statistics Canada’s Canadian Socio-economic Information Management (CANSIM) database. This is a time period of relative prosperity in Canada. Canada, as a whole, experienced significant economic growth from 2002–2008. We also rely on the male and female suicide rates to differentiate gender impacts of unemployment. The panel sets contained a total of 90 observations.

**Figure 1:**
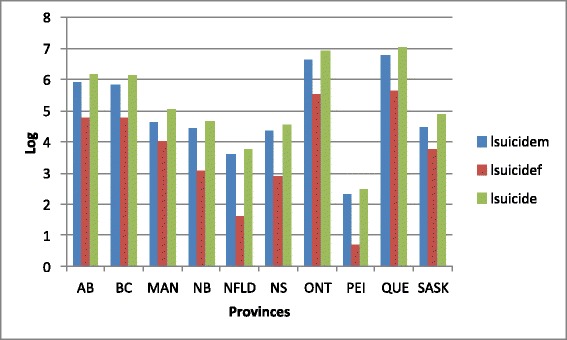
**Distribution of suicides across Canada, 2008.**

**Figure 2:**
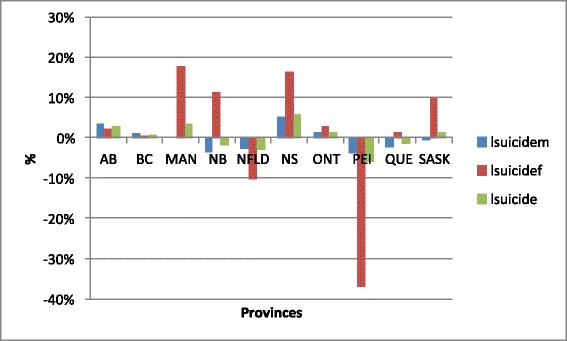
**Growth rates of suicides across Canada, 2000–2008.**

Figure 1 plots the natural logarithm of total, male and female suicide (lsuicide, lsuicidem, and lsuicidef, respectively) in the last year available, 2008, for the 10 Canadian provinces. Clearly evident from Figure 1 is that the suicide rate is far from uniform across the Canadian landscape. If any pattern is present, the suicide rates (total, male, and female) are all highest in the wealthier provinces (Ontario, Quebec, British Columbia, and Alberta) and lowest in the less-wealthy provinces that also tend to have the highest levels of unemployment (New Brunswick, Newfoundland, Nova Scotia, and Prince Edward Island (PEI)). Figure 2 presents the 2000–2008 growth rates that do not have any such pattern as indicated in Figure 1. Not only does PEI have the lowest rate of suicide in 2008, but has also had the most significant decreases in the rates of suicide, 2000 – 2008—Newfoundland also has experienced a notable drop in its suicide rates. The greatest growth rates are occurring in Manitoba, New Brunswick, Nova Scotia, and Saskatchewan.

Table 1 summarizes our main variables, either as natural logarithms, rates, or percentages. These summary statistics are calculated using the entire panel, with provincial-level summary statistics available to the interested reader from the authors.

**Table 1:** **Summary statistics**

**Variable**	**Mean**	**Std. Dev.**	**Min**	**Max**
**Suicide**	5.12	1.37	2.07	7.17
**Social Conditions (SC)**				
Alcohol sales	6.12	0.11	5.91	6.42
Female labor participation	60.67	3.59	50.5	67.6
Divorce rate	7.01	8.19	0.25	27.51
Unemployment rate	8.02	3.33	3.4	16.6
**Economic Conditions (EC)**				
Gini Index	0.41	0.02	0.36	0.44
GDP per Capita	10.48	0.22	10.15	11.09
GDP Growth	0.03	0.04	−0.06	0.21
Low income	11.88	1.45	8.85	14.11

### Empirical approach

In our subsequent analyses, we employ a number of different econometric methods. The point of a varied number of econometric methods is to investigate the potential impact of these different methods on the results. If, for example, the same qualitative results emerge regardless of the econometric method we can conclude that the results are robust. If, however, the results are sensitive to the particular econometric methods, we can argue that the econometric method must be chosen with caution because that choice will likely impact the qualitative nature of the results.

The empirical analysis begins by estimating the following reduced-form equation, using regressions with fixed effects:

1 $$ {s}_{it}={\alpha}_i+{\alpha}_t+{\beta}_0X{\hbox{'}}_{it}+{\varepsilon}_{it} $$

where *s*_*it*_ is a measure of suicide of province *i* on year *t*, measured by the log of the total number of suicides (for total, male and female subsamples)^d^; *α*_*i*_ is a set of province effects absorbing the effect of initial conditions; *α*_*t*_ is a set of year effects absorbing the effect of common trend. The control variables in *X* ^'^_*it*_ translate social and economic factors that may affect the number of suicides. Our set of regressors is composed of: the log of GDP per capita, GDP growth, unemployment rate, female labor participation rate, divorce rate, log of alcohol sales per capita, share of low income people, total immigration rate, dependency ratio.

Our primary intention in this paper is to identify the strength of these effects, but the reduced-form approach of these regressions may naturally be subject to criticisms despite being a common practice in the related literature on the economics of suicide.

In addition to running (1) with each regressor included individually in the equation we also group the variables into 3 self-explanatory blocks, as described below, and organized in Table 1. We use static Principal Component Analysis (hereafter PCA) to obtain the common factor(s) of each block of variables.

- *Social Conditions (SC)*: we use the following variables to construct the index: dependency ratio, alcohol consumption per capita (in 2002 USD), divorce rate and female labour participation rate. Only the first principal component was retained^e^.
- *Economic Conditions (EC)*: we use the following variables to construct the index: GDP per capita, the Gini coefficient and the low income percentile. Only the first principal component was retained^f^.

Given that the PCA is based on the classical covariance matrix, that is sensitive to outliers, we take one further step by basing it on a robust estimation of the covariance (correlation) matrix. A well suited method is the Minimum Covariance Determinant (MCD) that considers all subsets containing h% of the observations and estimates the variance of the mean on the data of the subset associated with the smallest covariance matrix determinant - we implement the Rousseeuw and van Driessen algorithm [54]. After re-computing the same indices with the MCD version we obtain, generally speaking, similar results after inspection of correlations, meaning that outliers are not driving our factor analysis. Table 2 provides the factor loadings and uniqueness.

**Table 2:** **Factor loadings and uniqueness**

	**Factors**		**Uniqueness**
**Variables**	**SC**	**EC**	
Alcohol sales	0.26		0.47
Dependency ratio	0.98		0.02
Female labor participation	0.26		0.46
Divorce rate	0.96		0.02
GDP per Capita		0.63	0.51
Gini index		0.93	0.13
Low income		0.89	0.21
% Explained	0.50	0.71	

We can interpret the principal components by focusing on the factor loadings onto them and the uniqueness of each variable. Given the relatively high uniqueness of alcohol sales and female participation, the social conditions factor (SC) essentially describes the divorce rate and dependency ratio of each province. Hence, we expect a positive sign of this factor in the regressions. Turning to the second block, the economic conditions (SC) are mainly described by distribution variables, to which corresponds the lowest uniqueness. Hence we also expect it to enter positively in the regressions.

### Treatment of endogeneity

The model described in the previous sub-section is in reduced-form and its results may be affected by endogeneity of some or possibly even all the covariates (as discussed in section 2 with respective to particular variables). Preliminary investigation revealed that the dependent variable was serially correlated such that we will use a dynamic panel approach that provides consistent estimates such as the General Method of Moments. One has still to decide whether to use as in Arellano and Bond [55] “difference-GMM” (DIF-GMM) or Arellano and Bover [56] “system-GMM” (SYS-GMM). These two approaches are not completely separate, because the SYS-GMM approach is actually an augmented DIF-GMM estimator [57,58] that uses potentially more information and internally available instruments in the estimation procedure. We have selected the “difference-GMM” approach in our case as a result of the following reasons: i ) SYS-GMM generates more internally available instruments, that is a one side of the coin since it can generate “too many instruments” (in a sense that many such instruments are “weak”) and so one needs to identify the “optimal” number of instruments in order to obtain efficient estimates [57], ii) The SYS-GMM also has one pragmatic disadvantage; this estimation technique is very complicated and one can easily get misleading results if the modelling procedure is not applied properly [57], iii) the SYS-GMM requires “the steady state” assumption throughout the analyzed period [57] and if it is not the case (i.e. if the lagged dependent variable does not converge towards the steady state levels), an important assumption of the SGMM is violated, iv) the SYS-GMM needs “more” observations to get “better” estimates, that is a limitation that especially applies to our case (we deal below with a sample of 90 observations, that is far from a large sample).

## Results and discussion

### Model selection

It is well known that the inclusion of particular control variables in a regression can wipe out (or change the signs of) any given bivariate relationship [59]. With these considerations in mind, prior to proper fixed-effects estimation, we employ the Bayesian Model Averaging (hereafter BMA) approach as a model selection method. Essentially, BMA treats parameters and models as random variables and attempts to summarise the uncertainty about the model in terms of a probability distribution over the space of possible models. The method is used to average the posterior distribution for the parameters under all possible models, where the weights are the posterior model probabilities. To evaluate the posterior model probability the BMA uses the Bayesian Information Criteria (BIC) to approximate the Bayes factors that are needed to compute the posterior model probability [60-62]. The output of the BMA analysis includes the posterior inclusion probabilities for variables and a sign certainty index.^g^ The higher the posterior probability for a particular variable the more robust that determinant for external capital flows appears to be.

Table 3 presents our results for the total, male and female total suicides and suicide rates. We observe that income per capita consistently shows up with a positive sign, while GDP growth has the opposite effect. As for distributional effects, the Gini index presents conflicting signs whereas the lowest income quintile unambiguously raises the propensity to commit suicide. Higher dependency ratio, divorce rate and female labor participation increase suicides but it depends whether we are looking at the aggregate variable or the gender split cases. Contrary to expectations, unemployment appears with a negative sign, but this may be the reflection of other unobserved factors that are correlated with suicide. Finally, immigration does not seem to matter for Canadian suicides. All in all, this analysis confirms, refutes or presents unclear evidence about the (prior) signs of several suicide determinants, in line with the discussion above.

**Table 3:** **Bayesian model averaging – determinants of suicide**

	**Suicide**		**Suicide (M)**		**Suicide (F)**		**Suicide rate**		**Suicide rate (M)**		**Suicide rate (F)**	
	**PIPs**	**Sign**	**PIPs**	**Sign**	**PIPs**	**Sign**	**PIPs**	**Sign**	**PIPs**	**Sign**	**PIPs**	**Sign**
Dependency ratio	1.00	+	1.00	+	0.85	-	0.03		0.02		0.25	
Unemployment rate	1.00	-	1.00	-	1.00	-	1.00	-	1.00	-	1.00	-
Female labor participation	1.00	+	1.00	+	0.00		0.00		1.00	+	1.00	+
Divorce rate	0.06		0.09		1.00	+	1.00	+	0.00		0.00	
GDP per Capita	1.00	+	1.00	+	1.00	+	1.00	+	1.00	+	1.00	+
GDP growth	1.00	-	0.14		1.00	-	1.00	-	1.00	-	1.00	-
Gini Index	1.00	+	1.00	+	1.00	+	0.58	-	0.53	-	0.94	-
Low income	0.09		0.99	+	0.00		1.00	+	1.00	+	1.00	+
Alcohol sales	0.72	+	0.00		1.00	+	1.00	+	1.00	+	1.00	+
Total migration	0.00		0.00		0.45		0.00		0.00		0.00	
R-squared	0.98		0.98		0.95		0.95		0.95		0.94	

Note: The dependent variable corresponds to different variants of suicide. The variables’ description is in the main text. The BMA analysis yields the posterior probabilities of inclusion (PIPs) and the sign certainty index of a relationship. A sign is given to the PIPs greater than 0.5. No sign means the sign of estimated relationship being uncertain.

### Panel VAR approach

We now use a Panel Vector Autoregression (PVAR) approach aimed at analysing the short-run transition of suicides to shocks to “fundamental” social and economic variables.^h^ It combines the traditional VAR approach, that treats all the variables in the system as endogenous, with the panel-data approach, that allows for unobserved individual heterogeneity. We specify a first-order VAR model as follows:

2 $$ {Y}_{i,t}={\Gamma}_0+\Gamma (L){Y}_{i,t}+{\nu}_i+{\varepsilon}_{i,t} $$

where *Y*_*i*,*t*_ is a vector of endogenous variables, *Γ*_0_ is a vector of constants, *Γ*(*L*) is a matrix polynomial in the lag operator, *ν*_*i*_ is a matrix of country-specific fixed effects, and *ε*_*i*,*t*_ is a vector or error terms (with zero mean and country-specific variance).

The main advantage of using a PVAR approach is that it increases the efficiency of the statistical inference, that would otherwise be suffering from a small number of degrees of freedom when the VAR is estimated at the country level. While this comes at the cost of disregarding cross-province differences by imposing the same underlying structure for each cross-section unit, Gavin and Theodorou emphasize that the panel approach allows one to uncover common dynamic relationships [63]. Moreover, by introducing fixed effects, *ν*_*i*_, one can allow for “individual heterogeneity” and overcome that problem. However, the correlation between the fixed effects and the regressors due to lags of the dependent variables implies that the commonly used mean-differencing procedure creates biased coefficients [64], that will be particularly severe if the time dimension is small [65]. This drawback can be avoided by a two-step procedure. First, we use the “Helmert procedure”, that is, a forward mean-differencing approach that removes only the mean of all future observations available for each country-year [55]. Second, we estimate the system by GMM and use the lags of the regressors as instruments, as the transformation keeps the orthogonality between lagged regressors and transformed variables unchanged [56]. In our model, the number of regressors is equal to the number of instruments. Consequently, the model is “just identified” and the system GMM is equivalent to estimating each equation by two-stage least squares.

Another issue that deserves attention refers to the impulse-response functions. Given that the variance-covariance matrix of the error terms may not be diagonal, we need to decompose the residuals so that they become orthogonal. We follow the usual Choleski decomposition of variance-covariance matrix of residuals, in that after adopting the abovementioned ordering, any potential correlation between the residuals of the two elements is allocated to the variable that comes first. In this vein, we experiment with our panel, where the ordering of variables for the system is as follows: *suicide, unemployment rate, divorce rate, low income, dependency ratio* and *alcohol sales*. ^i^The results of the system are presented in Table 4.

**Table 4:** **Variance decomposition of PVAR**

	**Suicide**	**Unemployment rate**	**Divorce rate**	**Low income**	**Dependency ratio**	**Alcohol sales**
**Suicide**	0.97	0.00	0.03	0.00	0.00	0.00
**Unemployment rate**	0.02	0.82	0.15	0.01	0.00	0.00
**Divorce rate**	0.00	0.03	0.94	0.02	0.00	0.00
**Low income**	0.02	0.06	0.86	0.06	0.00	0.00
**Dependency ratio**	0.07	0.13	0.01	0.01	0.78	0.00
**Alcohol sales**	0.02	0.01	0.02	0.01	0.03	0.92

Percentage of variation in the row variable explained by column variable.

Table 4 presents the variance decomposition of the variables included in the system, it is clear that the variance of each variable is essentially explained by itself, that bodes well for the inclusion of these variables as covariates in the main regressions. This is striking in the case of our main variable of interest, *suicide*, however in some other cases there are exceptions. Take the unemployment rate where own shocks explain 82% of the total variance, while divorce rate contributes with additional 15%. Or the low income percentile where own shocks account for 86% of the total variance and the unemployment rate with 6%.

The impulse-response functions (available to the interested reader from the authors) are far from elucidative. In fact, despite some relationships and signs are in accordance to prior expectations, overall, the confidence bands at the usual 5% level make the effects statistically insignificant in the case of shocks to suicides.

To conclude this section, the panel VAR does not offer suggestive evidence about the relation between suicide and social or economic variables. Hence, in order to get a clearer picture we need to embed our variables into a regression setting. We now turn to the results of the estimation of equation (1).

### Fixed effects panels

In this section we present the results of the estimation of equation (1) using fixed effects panel methods. Our results are displayed in Table 5.

**Table 5:** **Fixed effects regression (country + time effects)**

	**Suicide**	**Suicide (M)**	**Suicide (F)**	**Suicide rate**	**Suicide rate (M)**	**Suicide rate (F)**
Gini Index	−2.852	−2.720	−3.898	−2.585	−2.666	0.081
GDP per Capita	2.478***	1.784**	6.776***	0.211	0.225	−0.013
GDP growth	−2.500**	−2.914**	−1.434	−0.275**	−0.303**	0.028
Unemployment rate	0.081**	0.041	0.298**	0.026	0.018	0.008
Female labor participation	0.006	0.011	−0.040	−0.004	−0.003	−0.002
Divorce rate	0.001	0.032	−0.146	0.019	0.022	−0.003
Alcohol sales	−1.113**	−1.656*	0.845	−0.578***	−0.558***	−0.020
Low income	0.823**	0.924	0.800	0.223**	0.206**	0.018
Immigration rate	0.004**	0.003	0.014***	0.001	0.000	0.001**
Dependency ratio	10.030	5.024	46.741**	−3.333	−4.751	1.418
*Observations*	50	50	50	50	50	50
*R-squared*	0.644	0.442	0.787	0.445	0.475	0.422

Note: The dependent variable is the log of suicide and the suicide rate, for total population, male and female subsamples. All specifications include the estimate of a constant coefficient, not presented in this table for reasons of parsimony. Heteroskedastic-consistent standard errors are used for all inference, ***, ** and * denote significant coefficients, respectively at the 1, 5 and 10% confidence levels.

We can see that the divorce variable appears as statistically insignificant in all specifications irrespectively of the dependent variable under consideration [21]. However, there may be some unmeasured factors that are related to divorce and suicide rates. For instance, stress of depression may determine both suicide and marital dissolution. The coefficient on alcohol consumption per capita is statistically significant and negative for males. This negative coefficient may be the result of the effect of other unobserved factors that may be correlated with suicide rates and is not in accordance with prior panel data studies [21,22]. The impact of the unemployment rate on suicide is positive and significant meaning that unemployment increases suicide. No statistically significant impact of unemployment rates is found when using suicide rates as the dependent variable. This result is consistent with the finding from a panel data analysis in the US [66] but opposite to those results obtained by other research [22-24]. The estimate of the effect of GDP per capita is positive and statistically significant. A higher GDP per capita is associated with higher suicide mortality rates for both sexes. The coefficient estimates of the Gini index are negative and statistically insignificant. However, an insignificant positive effect is found in the study of for Germany [22]. Economic growth has a beneficial impact on suicide rates, as the coefficients for this variable are negative and significant. For both sexes, the coefficient of female labor participation rate is statistically insignificant [22,24,36].

If we run our fixed effects regression using the PCA variables *sc1* and *ec1* we obtain:

3 $$ {s}_{it}=0.293+0.125ec1+0.266sc1+0.008 unemp $$

(0.078) (0.031) (0.042) (0.006)

That is, both economic and social conditions seem to foster suicides, whereas we obtain a positive but statistically insignificant coefficient for the unemployment rate. Recall that according to the PCA’s factor loadings these positive effects are the ones we would expect in the present circumstances.

### Endogeneity – Arellano-Bond GMM estimation

To take into account possible endogeneity (and resulting bias and inconsistency of previous coefficient estimates) we also estimate the main equation (1) using Generalised Methods of Moments (GMM). An underlying advantage of the dynamic GMM estimation is that all variables from the regression that are not correlated with the error term (including lagged and differenced variables) can be potentially used as valid instruments [67]. As justified above we rely on the first-differenced GMM by Arellano and Bond [56]. The difference GMM treats the model as a system of equations in differences one for each time period (i.e. internal instruments are differenced variables). Looking at Table 6, results confirm our previous fixed-effect regressions and add some insights. Namely, total immigration now appears with a statistically significant positive coefficient, meaning that a migrant inflow increases the propensity to commit suicide. Also, the Gini index appears for the case of female suicides with a statistically negative coefficient.

**Table 6:** **Difference GMM estimates**

	**Suicide**	**Suicide (M)**	**Suicide (F)**	**Suicide rate**	**Suicide rate (M)**	**Suicide rate (F)**
Gini Index	−2.004	−0.208	−11.727**	−0.884	−1.015	0.131
GDP per Capita	2.371***	1.704**	6.463***	0.183	0.186	−0.003
GDP growth	−2.597***	−3.043***	−1.407	−0.188*	−0.228*	0.040
Unemployment rate	0.079***	0.015	0.423***	0.012	0.005	0.007**
Female labor participation	0.005	0.012	−0.033	−0.003	−0.002	−0.001
Divorce rate	0.001	0.013	−0.039	0.002	0.006	−0.004***
Alcohol sales	−1.402***	−1.882*	−0.068	−0.270*	−0.288*	0.018
Low income	0.798***	0.869**	0.977***	0.154**	0.137**	0.017
Immigration rate	0.003***	0.002	0.013***	0.001	−0.000	0.001***
Dependency ratio	11.147	15.159	1.551	4.169	2.128	2.042
*Observations*	40	40	40	40	40	40
*Hansen (p-value)*	1.000	1.000	1.000	1.000	1.000	1.000
*AR(1)*	0.136	0.164	0.110	0.051	0.115	0.121
*AR(2)*	0.144	0.161	0.257	0.097	0.056	0.296

Note: The dependent variable is the log of suicide and the suicide rate, for total population, male and female subsamples. Estimation is robust difference-GMM. Lagged regressors are used as suitable instruments. Robust heteroskedastic-consistent standard errors are used in all inference. The Hansen test evaluates the validity of the instrument set, i.e., tests for over-identifying restrictions. AR(1) and AR(2) are the Arellano-Bond autocorrelation tests of first and second order (the null is no autocorrelation), respectively. A constant term has been estimated but it is not reported for reasons of parsimony. *, **, *** denote significance at 10, 5 and 1% levels.

Results are also tested for their robustness by re-running (1) subject to the exclusion of one province at a time. These are presented in Table 7. When NFLD is excluded, GDP growth no longer leads to a fall in suicides. Also, when PEI is dropped the Gini index appears with a statistically significant negative coefficient. Alcohol consumption per capita reduces suicides when NB, NS and PEI are excluded from the sample. It interesting to note that when AB and QUE are dropped the percentile of people with low income ceases to matter. Finally, the lowest fit or R-square is found when NFLD is removed from the sample and the highest when SASK is removed. An obvious question to ask at this point is: why do the results change when certain provinces are removed from the analysis. This shows that there are particular provinces that are driving the results for particular relationships with suicide. Or, alternatively, because the sample size is not large, the addition of one province without a statistically significant relationship makes it appear as though there is no overall relationship. This latter possibility is likely behind the result for PEI and the Gini index. Inequality matters for most provinces in the context of suicide, but not for PEI; however, when PEI is added to the model that relationship disappears, on average. Consequently, these results must be taken into consideration when research is translated into health policy—what works for one sub-national unit may not work for another.

**Table 7:** **Fixed effects regression (country + time effects) (removing one province at a time)**

	**Province dropped**									
	**AB**	**BC**	**MAN**	**NB**	**NFLD**	**NS**	**ONT**	**PEI**	**QUE**	**SASK**
Gini Index	0.181	−2.193	−0.749	−3.231	−4.359	−3.285	−2.164	−9.127*	−2.481	−2.923
GDP per Capita	2.364***	2.450***	2.105***	2.328***	0.157	2.483***	2.479***	2.996***	2.376***	3.005***
GDP growth	−2.385*	−2.576**	−1.908**	−2.604**	−0.521	−2.504**	−2.681**	−2.611***	−2.578**	−3.219***
Unemployment rate	0.089***	0.086*	0.065	0.052	0.021	0.075**	0.069	0.083**	0.078*	0.093***
Female labor participation	−0.033	0.012	0.022	0.016	0.014	0.004	0.025	0.006	0.009	0.007
Divorce rate	0.009	−0.004	−0.003	0.015	0.024	0.003	0.030	−0.007	−0.004	−0.003
Alcohol sales	−0.983	−1.148	−0.964	−1.491*	−0.296	−1.077**	−1.437	−1.653**	−1.539	−0.895
Low income	0.648	0.789**	0.746*	1.012**	0.997***	0.853*	0.802*	0.487*	0.868	0.984**
Immigration rate	0.005**	0.004*	0.003	0.005**	0.005**	0.004*	0.009	0.003*	0.004**	0.004**
Dependency ratio	11.107	9.867	34.930**	15.094	−19.454*	10.407	10.405	−18.429	12.925	4.384
*Observations*	45	45	45	45	45	45	45	45	45	45
*R-squared*	0.671	0.649	0.684	0.673	0.599	0.645	0.649	0.749	0.655	0.753

Note: The dependent variable is the log of suicide and the suicide rate, for total population, male and female subsamples. All specifications include the estimate of a constant coefficient, not presented in this table for reasons of parsimony. Heteroskedastic-consistent standard errors are used in all inference, ***, ** and * denote significant coefficients, respectively at the 1, 5 and 10% confidence levels.

Following Gravelle et al., the models were also re-estimated including a squared economic inequality term to test for a non-linear relationship between inequality and rates of suicide [45]. A quadratic term for alcohol consumption has been included as well as a main effect as one might expect while a little drinking may reduce suicide risk, and a lot of drinking may increase it. In the first case, a significant negative effect was found in the squared Gini index coefficient, and the linear Gini index term itself got statistically positive (this non-linearity applies to the cases when total suicides and male suicides are the dependent variables). As to the second test, no significant effect was found in the squared alcohol consumption per capita term^j^.

Male and female suicides were also regressed on i) the lagged values of all explanatory variables and ii) the first difference of all explanatory variables (two different sets of regressions). Most coefficients lost their statistical significance with the few exceptions of unemployment, GDP growth and the low income percentile^k^.

## Conclusions

According to the World Health Organisation, and its member nations that report to it, there are approximately 3000 suicides each day, with the suicide rate having an increase of 60 percent since 1960 [68]. Given the rapid growth of the world’s population, this rate increase is troubling. This is particularly true because aggregate-level analyses of the suicide phenomenon have not established definitive results [19,68,69].

In this paper we empirically investigate the relationship between suicide and a number of socioeconomic variables with a panel of the 10 Canadian provinces, 2000 – 2008, using a variety of estimation methods. Though we do find some of the expected relationships between these variables, there is variation in the results depending on the type of estimation procedure employed. This is an important finding because most investigations into the determinants of suicide consider one estimation procedure. Consequently, one must ensure that the method is appropriate for the data and question at hand, and/or test the robustness of their results using more than one method of analysis. Moreover, it is clear that the relationships between suicide and socioeconomic variables are not constant for total, male, and female (logged) counts and rates; this provides more support for this separation. Also, particular provinces appear to be driving the results for certain socioeconomic variables. This result has important policy implications because any nationally-based suicide prevention policy may not have the desired outcome. As such, further research into this result is necessary if we are to have better informed suicide prevention. The primary purpose of this paper was not to test a particular theory. Rather, the purpose of this paper is empirically driven to flesh out the various suicide relationships using recent panel econometric techniques. We show that the relationships are far from monolithic and, generally, vary by gender.

Aside from the above-mentioned future research, there are also a number of other avenues future research should consider. There is the obvious call for the replication of these results in other contexts, both in terms of places and times. Moreover, though only annual aggregate data were available to us for the current analysis, monthly or quarterly time series data may prove to be instructive as in the cases of Stack [70] and Classen and Dunn [34]. Lastly, a necessary extension of this work would be to analyse more recent data to include the most recent economic downturn—unfortunately not available for Canadian provinces at the time of data gathering. Such an analysis would add yet another significant economic downturn and corresponding changes to socioeconomics providing more insight into the relationship between suicide and its various predictive factors.

## Endnotes

^a^Another approach used by a number of scholars considers an “option value” that depends on the prospects of life conditions improving in the future [10-12].

^b^English-Welsh, American, Scottish, Irish, German, Italian, Portuguese, Other foreign born, and Native born.

^c^The Canadian territores (Yukon, Northwest, and Nunavut) are excluded from the analysis because of their populations. This is a common practice in Canadian research; the low magnitude populations make most variables rather volatile over time and, arguably, unreliable for cross-sectional analyses.

^d^We will also use the suicide rate defined as total suicides divided by the population.

^e^A likelihood ratio (LR) test was used to examine the “sphericity” case, allowing for sampling variability in the correlations. This test comfortably rejects sphericity at the 1% level. The first factor explains 50% of the variance in the standardized data.

^f^A likelihood ratio (LR) test was used to examine the “sphericity” case, allowing for sampling variability in the correlations. This test comfortably rejects sphericity at the 1% level. The first factor explains 71% of the variance in the standardized data.

^g^For posterior inclusion probabilities greater than 0.50, a sign certainty index is presented, clearly suggesting the relationship being either positive or negative.

^h^We thank Inessa Love for providing her original code that was then adapted to our own purposes.

^i^Changing the ordering of the variables does not have a significant impact on the results.

^j^Results are not presented for reasons of parsimony but they are available from the authors upon request.

^k^Cf. footnote 11.
